# Green Pleural Effusion in a Patient With Hodgkin Lymphoma

**DOI:** 10.7759/cureus.67668

**Published:** 2024-08-24

**Authors:** Andres M Gutierrez-Gamez, Galyna Ivashchuk, Aristides Armas, Paula V Sainz Zuniga, Horiana Grosu

**Affiliations:** 1 School of Medicine, Tecnologico de Monterrey Escuela de Medicina y Ciencias de la Salud, Monterrey, MEX; 2 Pulmonary Medicine, MD Anderson Cancer Center, Houston, USA

**Keywords:** thoracentesis, green pleural effusion, lymphadenopathy, pleural effusion, lymphoma

## Abstract

A 35-year-old man presented with fever, drenching sweats, severe shoulder pain, and bilateral cervical and supraclavicular lymphadenopathy on physical exam. Computed tomography of the chest showed hilar, mediastinal, and supraclavicular adenopathy, multiple pulmonary nodules, and a left-sided pleural effusion. Thoracentesis revealed a green pleural effusion. After a systematic workup and core biopsy analysis of a supraclavicular lymph node, the patient was diagnosed with Hodgkin lymphoma. The green pleural effusion fluid was attributed to increased pleural fluid viscosity rarely seen in patients with lymphoma.

## Introduction

Hodgkin lymphoma, a relatively rare malignant B-cell neoplasm, represents 11% of all lymphomas in the United States [1]. Most patients with lymphoma present with rapidly enlarging mediastinal masses that invade adjacent thoracic structures. These patients usually have a short clinical history and exhibit signs and symptoms related to local invasion or compression of airways and adjacent vasculature [2]. Some patients present with pleural effusion, and these effusions have a relatively lower cytological yield compared with other solid tumors such as adenocarcinoma of the lung. Thus, the cause of effusion is more likely to remain undiagnosed after initial pleural fluid analysis. Diagnosing Hodgkin lymphoma based on clinical presentation and imaging studies is challenging because other etiologies have similar clinical presentations. Tuberculosis, nontuberculous *Mycobacterium* infections, and fungal infections may cause isolated mediastinal lymphadenopathy and pleural effusion and should be considered in the proper clinical setting [3].

## Case presentation

A 35-year-old man with a recent history of infectious mononucleosis five months prior presented with complaints of fever, drenching sweats, and severe pain in his left scapular region. He had frequent exposure to farm animals as well as wild animals given that he was a hunter.

**Figure 1:**
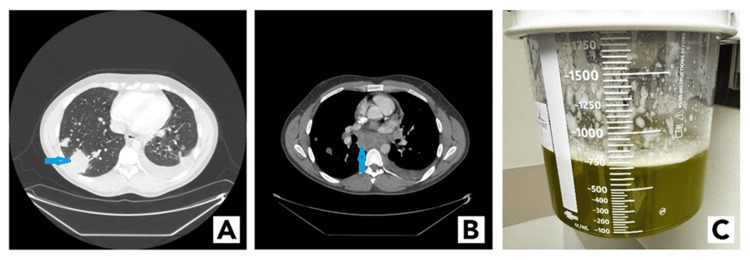
(A) Computed tomography of the chest, lung windows depicting a right lower lobe lung nodule (blue arrow) and a left-sided pleural effusion. (B) Computed tomography of the chest, mediastinal windows depicting a large subcarinal lymph node (blue arrow). (C) Green pleural effusion obtained from the patient.

A physical exam revealed bilateral cervical and supraclavicular lymphadenopathy. Subsequently, he was admitted for workup for lymphoma owing to his B symptoms. Computed tomography images of the patient's chest showed bulky multicompartment adenopathy; numerous pulmonary nodules and masses, including cavitary lesions with bronchograms; and a left-sided pleural effusion (Figure 1A, 1B, respectively). Given these findings and the patient's symptoms, our differential diagnosis included fungal infection, mycobacterial infection, and malignancy.

A left thoracentesis was performed to determine the etiology of the pleural fluid. A total of 800 mL of green pleural fluid was sent for analysis (Figure 1C). Pleural fluid analysis demonstrated an exudate, which was negative for cancer and infectious etiologies.

Next, we completed a bronchoscopy with endobronchial ultrasound and transbronchial needle aspiration (EBUS-TBNA) of the subcarinal and right hilar lymph nodes. The rapid on-site evaluation showed lymphocytes and no other abnormalities. In addition, the rapid on-site evaluation of the EBUS-TBNA analysis of a left lower lobe nodule showed no evidence of malignancy. Given these findings, a percutaneous ultrasound-guided core needle biopsy of a left supraclavicular lymph node was performed during the same procedure.

The final pathologic testing of the left supraclavicular lymph node demonstrated a syncytial variant of classical Hodgkin lymphoma, while the EBUS-TBNA samples were non-diagnostic of lymphoma.

## Discussion

This case illustrates the broad differential diagnosis for a patient who presented with pleural effusion, lymphadenopathy, and lung nodules. Because the patient had notable exposure to farm and wild animals, zoonotic diseases such as *Bartonella* and *Brucella* infections and Q fever were included in the differential diagnosis. In addition, whereas positivity for the Epstein-Barr virus and symptoms of fever, lymphadenopathy, hepatosplenomegaly, and fatigue are classic indications of mononucleosis, experiencing symptoms related to this infection five months after the initial presentation is unlikely. In cases such as this one, malignancy should be highly considered. 

One complication of an Epstein-Barr virus infection is an increased risk of Hodgkin lymphoma. However, in this case, in situ hybridization for the encoding region for Epstein-Barr virus was negative [4].

This case also emphasizes the importance of rapid on-site evaluation for patients undergoing bronchoscopy with endobronchial ultrasound, especially when lymphoma is a part of the differential diagnosis, because the negative results of the rapid on-site evaluation of the lymph node and fine-needle aspiration of the lung nodule in our case prompted us to complete a core biopsy analysis of the supraclavicular lymph node. Bronchoscopy with endobronchial ultrasound is more sensitive for de novo lymphoma (67%) [5], than for any other lymphoma.

Finally, the most interesting finding was the presence of the green left pleural effusion. The differential diagnosis for the cause of green effusion is broad and includes bilothorax, empyema, chylothorax, and cancer. In cases of bilothorax, bile leaks into the pleural space. Bilothorax is often secondary to trauma, surgery, or severe cholecystitis. Another cause for the green pleural effusion is *Pseudomonas aeruginosa* infection as it can produce a green pigment, pyocyanin, which can color the pleural effusion green. Our patient, however, did not have evidence of bilothorax nor infection. Hence, increased pleural fluid viscosity owing to cancer was considered the likely cause of the green pleural effusion [6,7]. Even though cytology results were negative for malignancy in the present case, malignancy was the most likely cause of the green pleural effusion given the low sensitivity of positive cytology on pleural effusion [8].

## Conclusions

This case illustrates a rare presentation of green-colored pleural effusion associated with Hodgkin lymphoma. In addition, although EBUS-TBNA has been established as an effective modality to diagnose carcinoma, its sensitivity for the diagnosis of lymphoma has been demonstrated to be lower, especially with Hodgkin lymphoma. This case highlights the challenge in diagnosing Hodgkin lymphoma and the need for multiple diagnostic tools to arrive at this diagnosis. One important aspect is recent advances in diagnostic approaches such as the use of rapid on-site evaluation that can help intraprocedural decision-making, such as in this case, where the clinician can adapt the procedure based on the results from the rapid on-site evaluation.
